# The Haematological Effects of Oleanolic Acid in Streptozotocin-Induced Diabetic Rats: Effects on Selected Markers

**DOI:** 10.1155/2019/6753541

**Published:** 2019-11-19

**Authors:** Charity M. Baloyi, A. Khathi, Ntethelelo H. Sibiya, Phikelelani S. Ngubane

**Affiliations:** ^1^Department of Health Sciences, Discipline of Human Physiology, University of KwaZulu-Natal, 4000, South Africa; ^2^Department of Pharmacy, Discipline of Pharmacy, University of Rhodes, 6140, South Africa

## Abstract

**Background:**

Sustained hyperglycaemia leads to the development of haematological alterations which, if left untreated, is associated with cardiovascular complications. Insulin is the mainstay drug in type 1 diabetes mellitus (T1D); however, the use of insulin is associated with haematological alterations that could further worsen cardiovascular complications. Therefore, the aim of the study was to investigate the haematological effects of oleanolic acid (OA) in streptozotocin- (STZ-) induced diabetic rats.

**Methods:**

The animals were separated into five groups; the nondiabetic group (ND), the diabetic control group (DC), and the treatment groups of insulin (170 *μ*g/kg, s.c), metformin (500 mg/kg, p.o), and OA (80 mg/kg, p.o). OA was administered orally twice a day. Thereafter, the animals were sacrificed, and blood and tissues were collected for haematological, hormonal, and oxidative status analysis.

**Results:**

Untreated diabetic rats exhibited hyperglycaemia, elevated glycated haemoglobin (HbA1c), oxidative stress, and a reduced erythropoietin (EPO) concentration when compared to ND rats. However, administration of OA attenuated hyperglycaemia, HbA1c, and EPO concentrations compared to DC rats. The reduction of blood glucose concentration, HbA1c, and improved EPO concentrations was further associated with a notable increase in red blood cell (RBC) count and other RBC indices. We also observed an increase in the antioxidant status of the RBCs with a concomitant decrease in oxidative stress.

**Conclusion:**

These findings suggest that OA improves diabetes-induced haematological changes caused by hyperglycaemia and attenuates the progression of cardiovascular complications in DM individuals.

## 1. Introduction

Sustained hyperglycaemia, as seen in type 1 diabetes, has been shown to induce cardiovascular complications through haematological alterations which include reduced deformability of the erythrocyte and increased haemolysis which is further correlated with reduced red blood cell (RBC) count, haemoglobin (Hb) concentration, haematocrit (Hct) levels, mean corpuscular haemoglobin concentration (MCHC), mean corpuscular volume (MCV), and red blood cell distribution width (RDW) [1–3]. The erythrocyte membrane plays an essential role in the regulation of surface deformability and flexibility [4, 5]. Hyperglycaemia has been shown to induce modifications and impairment of the erythrocyte membrane via increased production of reactive oxygen species (ROS). ROS causes nonenzymatic glycosylation of the RBC membrane proteins and the inactivation of RBC membrane antioxidant enzymes such as superoxide dismutase (SOD) and glutathione peroxidase (GPx) [6, 7]. ROS such as hydrogen peroxide (H_2_O_2_) cross the erythrocyte membrane and oxidize heme proteins, further decreasing the erythrocyte deformability and increasing osmotic fragility of the erythrocyte [8, 9]. RBC alterations have also been shown to induce rapid initiation of apoptosis. Apoptosis causes increased translocation of membrane phosphatidylserine (PS) from the inner side of the plasma membrane to the outer surface as detected by staining with fluorescein isothiocyanate- (FITC-) conjugated annexin-V [10–12]. The decreased deformability and increased haemolysis caused by ROS and apoptosis further decrease the oxygen-carrying capacity of the cells [8]. Furthermore, the prolonged reduction in oxygen-carrying capacity results in the development of diabetic anaemia [13]. Diabetic anaemia causes a reduction in RBC viscosity and increases aggregation and agglutination within the blood vessels, which consequently increases the formation of atherosclerotic plaque [2]. In addition, diabetic nephropathy caused by ongoing hyperglycaemia results in the destruction of the renal interstitium which is composed of interstitial peritubular fibroblast, further causing the impairment in erythropoietin (EPO) production [2, 13]. The impairment of EPO is further associated with a decrease in the production of RBC by the bone marrow and consequently diabetic anaemia [14].

Since complications are caused by sustained hyperglycaemic conditions, treatment is aimed at lowering blood glucose [15]. Insulin is currently the mainstay treatment for DM in addition to other classes of drugs [16]. However, the administration of insulin increases blood viscosity, causes agglutination, and promotes atherogenesis with prolonged use [17]. Metformin has also been shown to play a role in the progression of anaemia through the malabsorption of vitamin B_12_ [18]. Furthermore, the inability of conventional drugs to fully manage diabetes and its complications has led to the development of alternative approaches. The World Health Organization (WHO) has encouraged the use of medicinal plants as an alternative approach for the management of diabetes [19–21]. Pentacyclic triterpenes such as ursolic acid (UA) and maslinic acid (MA) are widespread throughout the plant kingdom and possess a broad spectrum of pharmacological properties which include hypoglycaemic, renoprotective, and antioxidant properties [22, 23]. Of interest in this study is oleanolic acid (OA), a triterpenoid related to MA and UA. OA is a pentacyclic triterpenoid that is widely spread in various plants such as *Momordica balsamina* and *Syzygium aromaticum* [16, 24, 25]. Previous studies in our laboratory have demonstrated the antihyperglycaemic, renoprotective, and antioxidant properties of OA in STZ-induced type 1 rat models [22, 25]. However, the haematological effects of OA in STZ-induced diabetic rats have not yet been established. Therefore, the aim of the study was to investigate the haematological effects of OA in STZ-induced diabetic rats.

## 2. Materials and Methods

### 2.1. Drugs and Chemicals

Chemicals and drugs were sourced as follows: streptozotocin (Sigma-Aldrich Chemical Company, Missouri, St. Louis, USA); metformin and insulin (Sigma-Aldrich Chemical Company, St. Louis, USA); dimethyl sulfoxide (DMSO), citric acid, sodium citrate, FITC annexin-V, and BD Falcon round-bottom tubes (BD Biosciences, San Jose, CA); sulphuric acid (BDH Chemicals LTD, Poole, England); and ELISA kits (Biocom Africa, South Africa).

### 2.2. Isolation of Oleanolic Acid (OA)

The extraction of OA was performed in a Chemistry laboratory at UKZN Pietermaritzburg campus. OA was isolated from *Syzygium aromaticum* [(Linnaeus) Merrill & Perry] [Myrtaceae] flower buds using a previously validated standard protocol that has been reported from our laboratories [26].

### 2.3. Animals

We used 30 male Sprague-Dawley rats (250-300 g, *n* = 6) which were bred and housed in the Biomedical Research Unit (BRU) in the University of KwaZulu-Natal. Standard laboratory conditions included constant temperature (22 ± 2°C), CO_2_ content of <5000 p.m., relative humidity of 55 ± 5%, and illumination (12 h light/dark cycles) with noise levels of less than 65 decibels. The animals were given rat chow daily for the 5-week experimental period. The institutional guidelines of the University of KwaZulu-Natal (AREC\041\018M) were used for the conduction of animal care. The animals were acclimatized for 5 days in metabolic cages before commencement of the study.

### 2.4. Induction of Diabetes

Type 1 DM was induced by a single intraperitoneal injection of 60 mg · kg^−1^ p.o STZ in freshly prepared 0.1 M citrate buffer (pH 6.3) following a previously described protocol [26].

### 2.5. Experimental Design

The short-term effects of OA were monitored for haematological parameters in separate groups of untreated and treated STZ-induced diabetic male Sprague-Dawley rats for 5 weeks using a previously validated standard protocol that has been reported from our laboratories [27]. Briefly, OA (80 mg/kg p.o) was administered twice every third day at 09:00 and 15:00 in separate groups of rats by means of a bulbed steel tube. The animals were divided into 5 groups: nondiabetic animals which served as the absolute control, untreated diabetic control group which received saline (saline 3 mL/kg p.o) to serve as negative control, 3 treatment groups of OA (80 mg/kg p.o), metformin (500 mg/kg p.o), and insulin (170 *μ*g/kg s.c), serving as positive controls, respectively. Over the period of 5 weeks, blood glucose concentrations were monitored where blood was collected via the tail prick method every 3^rd^ day at 09:00 am for the duration of experimental period using the Elite® glucometer (Elite (Pty) Ltd., Health Care Division, South Africa). EDTA anticoagulated blood was collected into the capillary tubes, which were then sealed at one end with plasticine, and centrifuged at 3000g for 5 min, after which RBC indexes in the capillary tubes were read using a microhaematocrit reader.

### 2.6. Terminal Studies

Blood and Tissue: at the end of the 5-week experimental period, all animals were sacrificed via exposure to halothane via an anaesthetic gas chamber (100 mg · kg^−1^) for 3 minutes (Biomedical Resource Unit, UKZN, Durban, South Africa). Thereafter, precooled heparinized containers were used for the collection of blood via cardiac puncture and centrifuged (Eppendorf centrifuge 5403, Germany) at 4°C, 503g for 15 minutes. In addition, the remaining RBCs were stored in separate Eppendorf tubes at -80°C in a BioUltra freezer until use for flow cytometry analysis. Kidney tissues were removed and weighed before freezing in liquid nitrogen. For further biochemical analysis, the kidneys were stored at −80°C in the BioUltra freezer (Snijders Scientific, Netherlands).

### 2.7. Haematological Analysis

100 *μ*L of blood was collected by cardiac puncture into individual precooled heparinized containers to assess the effects of OA on RBC count, MCV, MCHC, and haematocrit using a haemocytometer. In addition, the collected blood was used to determine RBC apoptosis using flow cytometry.

### 2.8. Biochemical Analysis

#### 2.8.1. Malondialdehyde (MDA) Assay

To investigate oxidative stress, 50 mg kidney tissues were homogenized in 500 *μ*L of 0.2% phosphoric acid; thereafter, tissues were centrifuged at 400 × g for 10 min. 400 *μ*L of the homogenate was supplemented with 400 mL 2% phosphoric acid and then separated into two glass tubes, each receiving equal volumes of the solution. To ensure an acidic pH of 1.5, 200 *μ*L of 1 M HCl was added to the sample and blank test tubes. Both solutions were heated at 100°C for 15 min and allowed to cool at room temperature. 96-well microtiter plates were used to check the absorbance at 532 nm on a BioTek mQuant spectrophotometer (BioTek, Johannesburg, South Africa). The absorbance from these wavelengths was used to calculate the concentration of MDA using Beer's Law.

(1) $$\begin{array}{c} \text{Concentration of MDA }\left(\text{mM}\right)=\frac{\text{Average Absorbance}}{\text{Absorption coefficient }\left(156\text{ mmo}{\text{l}}^{-1}\right)}. \end{array}$$

#### 2.8.2. Superoxide Dismutase (SOD), Glutathione Peroxidase (GPx), Erythropoietin (EPO) Concentrations, and HbA1c

SOD, GPx, EPO, and HbA1c concentrations were analyzed using separate specific ELISA kits (Elabscience and Biotechnology, Wuhan) that use the Sandwich-ELISA method, strictly following manufacturer's instruction.

### 2.9. Annexin-V Quantification

To acquire data, the BD FACS Canto-II flow cytometer and BD FACSdiva software (BD Biosciences, San Jose, CA) were used following a previously described protocol [27].

**Figure 1:**
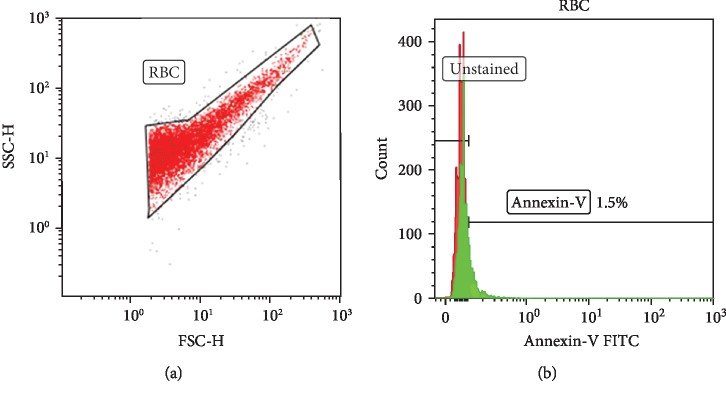
The gating strategy for annexin-V expression. The gating strategy applied. (a) The colour dot plot represents the RBCs based on forward scatter (FSC) and side scatter (SS). (b) The expression of annexin-V.

Detector settings: for small particle detection, forward scatter (FS) and side scatter (SS) parameters were set at a log scale (Figure 1(a)). For optimal antibody concentration detection, antibody titration assays were performed. Apoptotic RBC level measurement: annexin-V FITC was used (Figure 1(b)); annexin-V binds to the translocated phosphatidylserine (PS).

### 2.10. Statistical Analysis

Data is expressed as means ± standard error of means (SEM). Statistical analysis was conducted using GraphPad Prism InStat Software (version 5.00, GraphPad Software, San Diego, California, USA). One-way and two-way analysis of variance (ANOVA) followed by the Tukey-Kramer post hoc test was used to analyze differences between the controls and the experimental groups. Values of *p* < 0.05 indicate statistical significance.

## 3. Results

### 3.1. Blood Glucose Concentration

**Figure 2:**
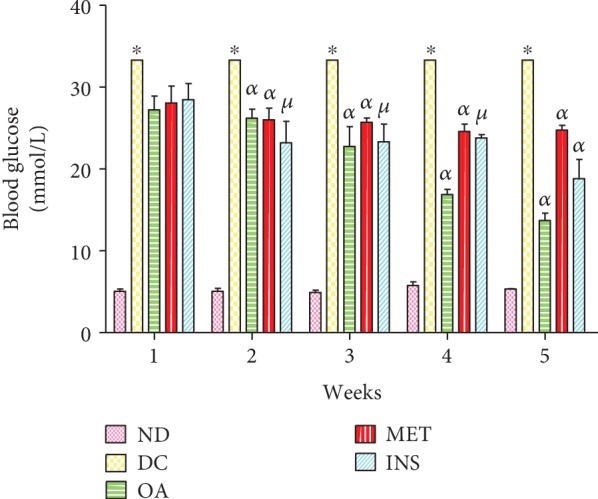
Blood glucose concentration of nondiabetic (ND), diabetic control (DC), and diabetic rats treated with OA, insulin, and metformin measured over a period of 5 weeks. Values are expressed as means ± SEM. ^∗^*p* < 0.05 by comparison with nondiabetic control. ^*α*^*p* < 0.05 by comparison with diabetic control. ^*μ*^*p* < 0.05 by comparison with OA-treated group.

Figure 2 shows blood glucose concentrations in nondiabetic (ND), diabetic control (DC), and diabetic rats treated with OA, insulin, and metformin over the 5-week period. The diabetic control group showed significantly increased glucose concentrations by comparison with nondiabetic control rats throughout the 5-week experimental period. Treatment with OA, insulin, and metformin decreased blood glucose concentrations from week 2 up to week 5 by comparison with untreated diabetic rats (*p* < 0.05; OA, MET, and INS vs. DC).

### 3.2. Effects on Haematological Parameters

Table 1 shows the haematological parameters of nondiabetic (ND), diabetic control (DC), and diabetic rats treated with OA, insulin, and metformin over the 5-week period. The diabetic control group showed a significant decrease in Hb concentrations, MCV, and Hct levels by comparison with nondiabetic animals. RBC, MCHC, RDW, and MCH of STZ diabetic rats decreased by comparison with nondiabetic rats, although this did not reach significance. Diabetic rats treated with OA however, together with insulin and metformin, showed an increase in RBC count, Hb concentrations, Hct levels, MCHC, and MCV by comparison with untreated STZ diabetic rats (*p* < 0.05; OA, MET, and INS vs. DC).

### 3.3. Effects on Plasma Erythropoietin Concentrations

**Figure 3:**
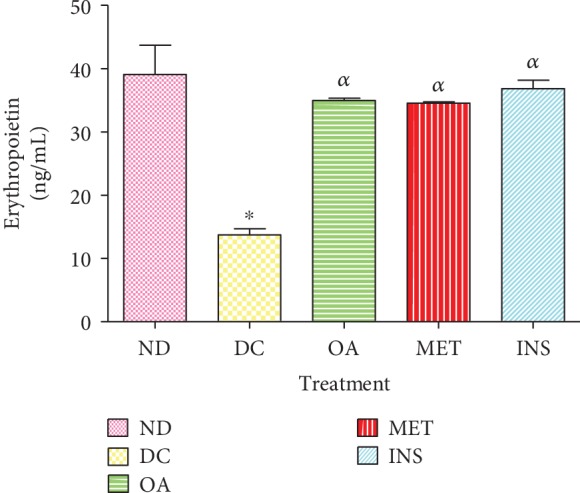
EPO concentrations in nondiabetic (ND), diabetic control (DC), and diabetic rats treated with OA, insulin, and metformin measured over a period of 5 weeks. Values are presented as means ± SEM. ^∗^*p* < 0.05 by comparison with nondiabetic control. ^*α*^*p* < 0.05 by comparison with diabetic control.

Figure 3 shows the comparison of EPO concentrations in nondiabetic (ND), diabetic control (DC), and diabetic rats treated with OA, insulin, and metformin over the 5-week period. Diabetic controls showed a significant decrease in EPO concentration by comparison with nondiabetic animals (*p* < 0.05, DC vs. ND). This was associated with a decrease in RBC count in diabetic rats (Table 1). Interestingly, the administration of OA, similarly to insulin and metformin, significantly increased EPO concentrations after 5 weeks of the experimental period. This was also associated with an increase in RBC count as shown in Table 1.

### 3.4. Effects on MDA, SOD, and GPx Concentrations

Table 2 shows the MDA, SOD, and GPx of nondiabetic (ND), diabetic control (DC), and diabetic rats treated with OA, insulin, and metformin measured over the 5-week period. The diabetic control group showed a significant increase in MDA concentration of both plasma and kidney tissues by comparison with nondiabetic animals. This was associated with a significant decrease in plasma SOD and GPx concentration by comparison with untreated nondiabetic (*p* < 0.05, DC vs. ND). Diabetic rats treated with OA significantly decreased MDA concentration in both plasma and kidney tissues by comparison with plasma and kidneys of untreated diabetic animals (*p* < 0.05, OA vs. DC). This was associated with an improvement of plasma SOD and GPx concentration by comparison with untreated diabetic rats.

### 3.5. Effects on Plasma Glycated Haemoglobin Concentration (HbA1c)

**Figure 4:**
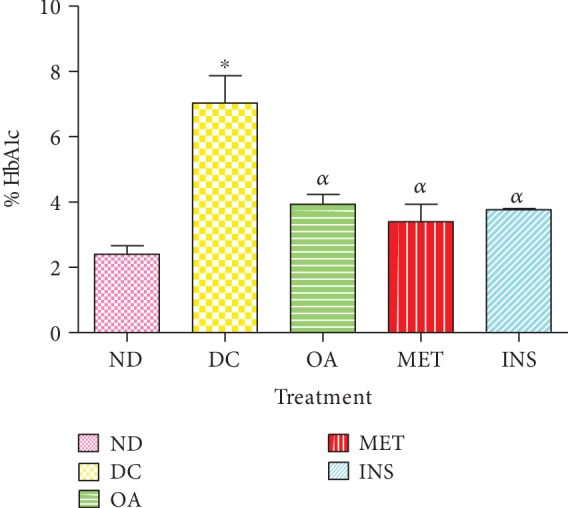
HbA1c concentration of nondiabetic (ND), diabetic control (DC), and diabetic rats treated with OA, insulin, and metformin over a period of 5 weeks. Values are presented as means ± SEM. ^∗^*p* < 0.05 by comparison with nondiabetic control. ^*α*^*p* < 0.05 by comparison with diabetic control.

Figure 4 shows the HbA1c in nondiabetic (ND), diabetic control (DC), and diabetic rats treated with OA, insulin, and metformin over the 5-week period. Diabetic controls showed a significant increase in HbA1c concentration by comparison with nondiabetic control (*p* < 0.05). The administration of OA, together with insulin and metformin, significantly decreased HbA1c concentration by comparison with the diabetic controls (*p* < 0.05).

### 3.6. Percentage of Annexin-V

**Figure 5:**
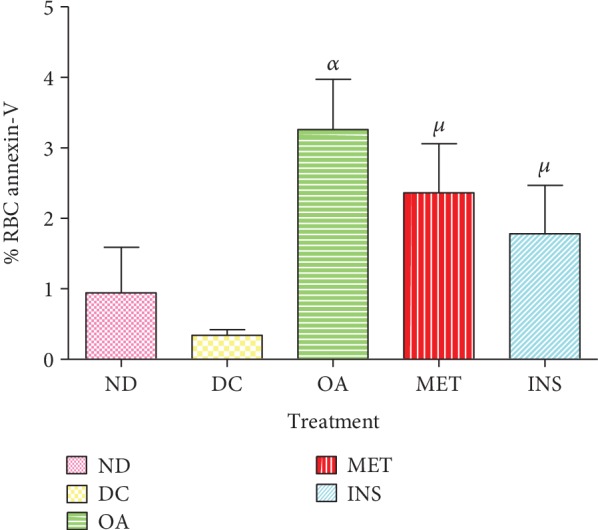
Annexin-V concentration of nondiabetic (ND), diabetic control (DC), and diabetic rats treated with OA, insulin, and metformin measured over a period of 5 weeks. Values are presented as means ± SEM. ^*α*^*p* < 0.05 by comparison with diabetic control. ^*μ*^*p* < 0.05 by comparison with the OA-treated group.

Figure 5 shows the annexin-V concentration of nondiabetic (ND), diabetic control (DC), and diabetic rats treated with OA, insulin, and metformin over the 5-week period. Diabetic controls showed a decreased RBC membrane annexin-V by comparison with the nondiabetic control group even though it did not reach statistical significance. This was associated with a significant decrease in MCV as shown in Table 1 by comparison with the nondiabetic control group (*p* < 0.05). The administration of OA however significantly increased RBC membrane annexin-V percentage by comparison with the diabetic control group (*p* < 0.05). This was also correlated with a significant increase in MCV as shown in Table 1 by comparison with the nondiabetic control group (*p* < 0.05).

### 3.7. Correlation between Mean Corpuscular Volume and Annexin-V

**Figure 6:**
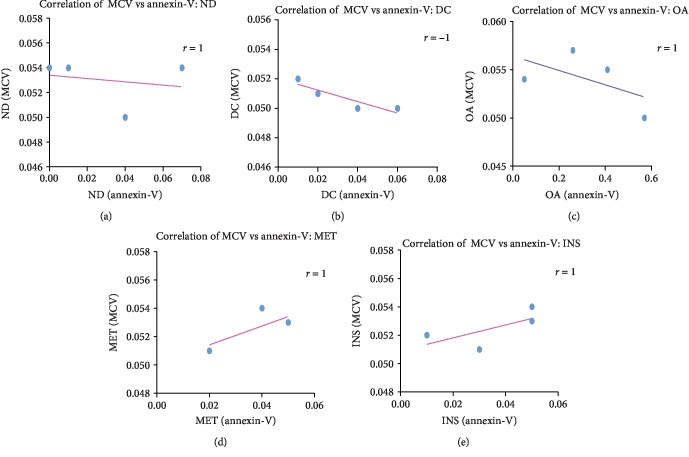


Figures 6(a) and 6(b) show the correlation between annexin-V and MCV in the nondiabetic control group (*r* = 1; *p* = 0.7) and the diabetic control group (*r* = −1; *p* = 0.01) of the STZ-induced diabetic animals. A significant decrease in annexin-V as seen in Figure 5 in the diabetic group is associated with a decrease in MCV by comparison with the nondiabetic group.

Figures 6(c)–6(e) show the correlation between annexin-V and MCV in animals treated with OA (*r* = 1; *p* = 0.7) and standard drugs, MET (*r* = 1; *p* = 0.3), and INS (*r* = 1; *p* = 0.3) of the STZ-induced diabetic animals. The OA group together with the standard drugs showed a significant increase in annexin-V and a significant increase in MCV in comparison with the diabetic control group.

## 4. Discussion

The present study investigated the haematological effects of OA in STZ-induced diabetic rats. STZ is widely used to establish diabetic animal models that mimic human diabetes [10]. In our laboratory, we have previously shown that 60 mg/kg of STZ selectively destroys pancreatic beta cells that secrete insulin, thereby producing a hyperglycaemic type 1 diabetic animal model [16, 25, 28, 29]. As confirmed by our current study, the diabetic control group showed a significant increase in blood glucose concentration, suggesting that STZ significantly destroyed the pancreatic beta cells in the diabetic control group. DM is a group of metabolic disorders characterized by hyperglycaemia which is associated with haematological alterations that progress into cardiovascular complications if left untreated [30].

Erythrocytes play an essential role in the delivery of oxygen to body tissues throughout the circulatory system [31]. RBCs can endure large continuous flow conditions along the vascular tree and narrow capillaries to deform without rupturing [4]. Furthermore, hyperglycaemia through the increase formation of ROS has been shown to impair RBC deformability, further causing an increase in RBC haemolysis [10, 12, 32]. The impairment in RBC deformability is correlated with haematological changes which include reduced RBC count, Hb concentration, Hct levels, MCV, MCHC, and RDW concentration as demonstrated by the hyperglycaemic STZ-induced diabetic animals in our study [33, 34]. Increased ROS formation causes nonenzymatic glycosylation of proteins on the erythrocyte membrane, cross-linking of membrane lipids, and inactivation of RBC antioxidant enzymes such as SOD and GPx [33]. STZ-induced diabetic rats showed increased MDA levels by comparison with nondiabetic animals which indicates, particularly, an increase in lipid peroxidation and oxidative stress [32]. The untreated hyperglycaemic STZ-induced diabetic rats also showed a significant increase in HbA1c which increases extracorpuscular Hb. An increase in corpuscular Hb has been shown to cause the reduction in RBC fragility, therefore a shortened RBC lifespan [6, 35]. OA however, together with insulin and metformin, significantly improved the haematological parameters. The ability of OA to significantly improve the haematological parameters may be due to OA's ability to decrease blood glucose and to improve antioxidant status. Previous studies in our laboratory have shown that OA decreases blood glucose concentration by increasing glycogen synthesis in the liver and skeletal muscle thereby improving glucose uptake by these tissues [16, 24, 36]. In addition, naturally occurring triterpenoids such as glycyrrhizic acid and ursolic acid have been previously reported to improve hyperglycaemic-induced haematological alterations by decreasing blood glucose concentration [37, 38]. OA may have also improved RBC count by improving the absorption of both vitamin B_12_ and folic acid, since vitamin B_12_ and folic acid play an essential role in red blood cell maturation [18]. Erythroblasts require folic acid and vitamin B_12_ for proliferation during their differentiation (39). Deficiency of both vitamin B_12_ and folic acid inhibits purine and thymidylate syntheses, impairs DNA synthesis, and causes erythroblast apoptosis further resulting in anaemia [18]. The animals treated with metformin however showed a decrease in RBC count in comparison with OA-treated animals which may be due to metformin's ability to cause the malabsorption of vitamin B_12_ and folic acid [18].

OA also improved the RBC indices possibly by improving the antioxidant status since previous studies have shown the ability of OA to improve SOD and GPx in STZ-induced diabetic rats [23]. SOD and GPx play a crucial role in the maintenance of haemoglobin in a decreased oxygen-binding form, in limiting oxidative modifications of membrane lipids, structural proteins, and metabolic enzymes, to keep the RBC alive during its lifespan [8]. SOD and GPx also detoxify ROS product to less toxic product, therefore slightly restoring RBC membrane integrity (40). In harmony with our study, in a previous study conducted by Jain et al., OA and UA were shown to ameliorate lipid peroxidation in STZ-induced rats by reducing the MDA production as also demonstrated in our study [37]. Another study has previously shown that OA increases the generation of antioxidant enzymes and the expression of oxidative stress-sensitive transcription factor NrF2 and MAO kinases, mainly extracellular signal-regulated kinase (ERK) and c-Jun N-terminal kinase (JNK) [38]. In addition, previous studies have reported that ROS induces apoptosis [12]. Apoptosis is a programmed cell death where unwanted cells are removed [11]. One of the earliest events of apoptosis includes translocation of membrane phosphatidylserine (PS) from the inner side of the plasma membrane surface to the outer surface [8]. Annexin-V, a Ca^2+^-dependent phospholipid-binding protein, has high affinity for PS, which can be detected by FITC fluorochrome-labeled annexin-V using flow cytometry [12]. Our current study showed a decrease of annexin-V in STZ-induced diabetic animals by comparison with nondiabetic rats. Surprisingly, treatment with OA aggravated the translocation of PS from the inner surface to the outer surface. Shuhua et al. demonstrated that OA induces activation of the intrinsic cell apoptosis pathway. MCV is the average size of the RBC; MCV decreases with increasing cell age [33]. Decreased MCV has been shown to be correlated with an increase in apoptosis as marked by an increase in annexin-V through osmotic resistance and shear stress [8]. Our current study showed a reduced MCV with a contaminant decrease in annexin-V in the OA-treated group; this might be due to OA's ability to promote apoptosis.

Renal dysfunction also caused by ROS through hyperglycaemia has been shown to be associated with a decrease in EPO concentration as interstitial fibroblasts responsible for EPO secretion are often destroyed during the development of diabetic nephropathy [20]. However, the administration of OA elevated EPO secretion by the kidneys possibly by overcoming the injury to peritubular interstitial fibroblast cells. The improvement in EPO secretion may also have accounted for the increased RBC count and other RBC indices. We speculate that the ability of OA to improve haematological changes may be associated with the improvement in cardiovascular hemodynamics, by decreasing RBC agglutination and aggregation.

## 5. Conclusions

Current drugs of DM are associated with cardiovascular complications through haematological alterations. There have been ongoing studies on the therapeutic beneficial effects of medicinal plants in individuals with cardiovascular complications. The current study demonstrates the haematological effects of OA in STZ-induced diabetic rats and therefore the improvement of cardiovascular complications with prolonged use. Our results showed that OA reduces hyperglycaemia, further improving erythrocyte function possibly through the increase in EPO secretion, improvement of antioxidant status, and decrease of glycated haemoglobin and by improving cell survival for the whole lifespan of the erythrocyte.

## Figures and Tables

**Table 1:** Haematological parameters in nondiabetic (ND), diabetic control (DC), and diabetic rats treated with OA, insulin, and metformin measured over a period of 5 weeks. Values are presented as means ± SEM.

Treatment					
Parameters measured	ND	DC	OA	INS	MET
RBC (×10 cells/*μ*L [3])	6.21 ± 0.50	5.70 ± 0.19	7.16 ± 0.72*^α^*	8.03 ± 0.35*^α^*	8.78 ± 0.11*^α^*
Hb (g/dL)	12.28 ± 0.63	9.49 ± 0.26^∗^	12.38 ± 0.25*^α^*	14.89 ± 0.85*^α^*	15.49 ± 0.54*^α^*
Hct (%)	38.58 ± 0.02	31.18 ± 0.47^∗^	38.20 ± 4.23*^α^*	41.34 ± 1.96*^α^*	43.73 ± 1.48*^α^*
MCHC (g/dL)	37.90 ± 0.65	36.10 ± 0.32	32.77 ± 0.60*^α^*	36.68 ± 0.22*^μ^*	37.88 ± 1.35^#^
MCV (fL)	53.00 ± 1.00	50.76 ± 0.25^∗^	53.67 ± 1.48*^α^*	52.50 ± 0.65	52.75 ± 0.63
MCH (pg)	20.08 ± 0.47	18.30 ± 0.00	18.19 ± 0.26	19.93 ± 0.72	19.98 ± 0.89
RDW (fL)	11.08 ± 0.13	10.67 ± 0.22	11.70 ± 0.30	11.70 ± 0.41	11.88 ± 0.30

^∗^ *p* < 0.05 by comparison with nondiabetic control. ^*α*^*p* < 0.05 by comparison with diabetic control. ^*μ*^*p* < 0.05 by comparison with the OA-treated group.

**Table 2:** MDA, SOD, and GPx concentrations in nondiabetic (ND), diabetic control (DC), and diabetic rats treated with OA, insulin, and metformin over a period of 5 weeks. Values are presented as means ± SEM.

Treatment					
Parameters measured	ND	DC	OA	INS	MET
Plasma MDA (*μ*mol/g protein)	0.90 ± 0.02	7.25 ± 0.06^∗^	2.20 ± 0.02*^α^*	2.89 ± 0.08	5.51 ± 0.02
SOD concentration (nmol min^−1^ mL^−1^ g protein)	3.50 ± 0.63	1.00 ± 0.27	3.30 ± 0.01*^α^*	3.00 ± 0.02*^μ^*	2.90 ± 0.03*^μ^*
GPx concentration (nmol min^−1^ mL^−1^ g protein)	1.44 ± 0.03	0.21 ± 0.04^∗^	0.79 ± 0.05*^α^*	1.67 ± 0.01*^αμ^*	0.83 ± 0.01*^α^*
Kidney MDA (*μ*mol/g protein)	0.39 ± 0.15	4.20 ± 0.12^∗^	1.10 ± 0.01*^α^*	1.10 ± 0.01*^α^*	1.27 ± 0.01^*α*^

^∗^ *p* < 0.05 by comparison with nondiabetic control. ^*α*^*p* < 0.05 by comparison with diabetic control. ^*μ*^*p* < 0.05 by comparison with the OA-treated group.

## Data Availability

The data used to support the findings of this study are available from the corresponding author upon request.
